# Distinct Genomic Aberrations between Low-Grade and High-Grade Gliomas of Chinese Patients

**DOI:** 10.1371/journal.pone.0057168

**Published:** 2013-02-22

**Authors:** Yunbo Li, Dapeng Wang, Lei Wang, Jinhai Yu, Danhua Du, Ye Chen, Peng Gao, Duen-Mei Wang, Jun Yu, Feng Zhang, Shuanglin Fu

**Affiliations:** 1 First Hospital of Jilin University, Changchun, PR China; 2 CAS Key Laboratory of Genome Sciences and Information, Beijing Institute of Genomics, Chinese Academy of Sciences, Beijing, PR China; 3 Norman Bethune College of Medicine, Jilin University, Changchun, PR China; 4 Jilin University, Changchun, PR China; 5 Northeast Normal University, Changchun, PR China; Beijing Tiantan Hospital, Capital Medical University, China

## Abstract

**Background:**

Glioma is a type of tumor that develops in the central nerve system, mainly the brain. Alterations of genomic sequence and sequence segments (such as copy number variations or CNV and copy neutral loss of heterozygosities or cnLOH) are thought to be a major determinant of the tumor grade.

**Methods:**

We mapped genomic variations between low-grade and high-grade gliomas (LGG and HGG) in Chinese population based on Illumina’s Beadchip and validated the results using real-time qPCR.

**Results:**

At the cytoband level, we discovered: (1) unique losses in LGG on 5q, 8p and 11q, and in HGG on 6q, 11p, 13q and 19q; (2) unique gains in the LGG on 1p and in HGG at 5p, 7p, 7q and 20q; and (3) cnLOH in HGG only on 3q, 8q, 10p, 14q, 15q, 17p, 17q, 18q and 21q. Subsequently, we confirmed well-characterized oncogenes among tumor-related loci (such as *EGFR* and *KIT*) and detected novel genes that gained chromosome sequences (such as *AASS*, *HYAL4*, *NDUFA5* and *SPAM1*) in both LGG and HGG. In addition, we found gains, losses, and cnLOH in several genes, including *VN1R2*, *VN1R4*, and *ZNF677*, in multiple samples. Mapping grade-associated pathways and their related gene ontology (GO) terms, we classified LGG-associated functions as “*arachidonic acid metabolism*”, “*DNA binding*” and “*regulation of DNA-dependent transcription*” and the HGG-associated as “*neuroactive ligand-receptor interaction*”, “*neuronal cell body*” and “*defense response to bacterium*”.

**Conclusion:**

LGG and HGG appear to have different molecular signatures in genomic variations and our results provide invaluable information for the diagnosis and treatment of gliomas in patients with variable duration or diverse tumor differentiation.

## Introduction

Glioma is a major tumor type that derives from glial cells of the central nerve system, including of spine and the brain. Gliomas are classified into four grades, from I to IV, with increasing exacerbation according to histology [1]. Alternatively, they are categorized into ependymomas, astrocytomas, oligodendrogliomas, and mixed gliomas, according to the cell types that are anatomically defined from different brain regions based on the brain topology [2]. Glioma is considered as low prevalence but an increasing detection probability due to enhanced early detection techniques and procedures, which include X-ray, magnetic resonance imaging (MRI), and computed tomography (CT) [3]. Patients with gliomas often have a morbid state in various degrees, including pain, epilepsy, mental disorder, visual disturbance, hearing impairment, insomnia, and nausea [4]. Traditional therapies for the tumors, such as surgery, radiotherapy, chemotherapy, and oral medication, are often individually or selectively cooperated to remit or to cure the symptoms according to patient’s condition and tumor grade [5], [6].

Although the degree of copy number variations (CNVs) among healthy human populations do vary, abnormal increase of CNVs and cnLOHs are thought to be at least one of the important causative factors for gliomas and other cancers [7], [8], [9]. Large-scale genomic aberration and gene expression studies on gliomas have lead to identifications of genes, which are involved in numerous cellular functions and metabolic pathways (*PCDH9*, *CXCL12*, *MYC*, *PDGFRA*, *PARK2*, *DMBT1*, *TOP2A*, *PTEN*, *ARF*, *TP53*, *P16*, *CDKN2B*, *RB1*, *EGFR,* and *NF1* [9], [10], [11], [12], [13], [14], [15], [16], [17]), as well as those related to neural development, cell signaling (RAS/RAF, RTK, MAPK, PI3K, and ROCK), and tumor suppression (p53 and RB) [1], [6], [11], [16], [18], [19], [20], [21], [22], [23], [24], [25], [26], [27], [28]. These genes serve as biomarkers useful for tumor classification, prognosis, and targeted therapies [17], [23], [29], [30], [31], [32], [33], [34].

Previous studies have been adding complexity for the understanding of gliomas at molecular levels. First, both nuclear and mitochondrial genomes show numerous structural variations [35]. Second, there are heterogeneous patterns of structural variations and/or expressions depending on the ways of classifying and grading gliomas, even between primary and secondary gliomas and between pediatric and adult gliomas [14], [20], [36], [37]. Third, consistency of the experimental results at DNA, RNA, and protein levels is poor due to complications of transcriptional and post-translational regulations, as well as enzymatic modifications [6], [12], [38], [39]. Fourth, relationship between sequence variations and prognosis are influenced by certain patient-specific parameters. For instance, older patients tend to have shorter survival time than younger patients [1], [40]. Fifth, co-variations can occur in different chromosomes and one gene may have multiple variations in different combinations [41], [42]. Last, relatively homogenous DNA variations have been found when topological locations (e.g., central vs. peripheral) are compared, where cross-contamination between normal and tumor tissues may contribute to the incorrect detection of structural variations [6], [43], [44]. Nevertheless, the existing literature related to genomic alterations and their molecular mechanisms in gliomas lacks information from Chinese populations.

Together with increasing throughput and efficiency of sequencing and microarray technologies for SNP and CNV discovery and typing, in-depth study on special tumor types become feasible, especially when two novel parameters–physical (Log R ratio) and genetic (B allele frequency) alterations/variability–are used to increase sensitivity and accuracy [45]. We report the first study in which we compared chromosomal aberrations of low-grade and high-grade gliomas (LGG and HGG) in Chinese populations to unveil differences of tumor-related sequence variations, including chromosomes and cytobands, and individual genes in the context of pathways and function categories.

## Materials and Methods

### Ethics Statement

This study was approved by the ethics committee at First Hospital of Jilin University, and all patients who agreed to this study signed their informed consents. In particular, some of the written informed consents were also obtained from the next of kin, caretakers, or guardians on the behalf of the minors/children participants involved in this study.

### Sample Preparation and Genotyping

18 gliomas, including nine of each for both low-grade (grade II) and high-grade (6 grade III and 3 grade IV) tumors (as LGG and HGG), as well as 18 paired blood samples were collected at First Hospital of Jilin University (Table 1). In general, patients with LGG (average age of 41) and HGG (average age of 47) have no prior history of chemotherapy or radiation treatment, and the tumors and matched peripheral blood samples were obtained at the time of tumor resection. The blood samples were immediately snap-frozen in liquid nitrogen and stored at −80°C before DNA extraction. Portions of the solid tumors were treated in the same way as the blood samples and others were paraffin-embedded for histopathology. The genomic DNA was isolated by using QIAamp DNA Mini Kit (QIAGEN Inc). DNA quality and purity were determined based on agarose gel electrophoresis and spectrophotometry. Four additional gliomas for RNA RT-qPCR validation were also collected at the same hospital and treated according to the same protocol as used for the other samples (Table 1). Genotyping was carried out by using Illumina Sentrix Human CNV370-Quad v3 BeadChip according to the manufacturer’s instruction.

**Table 1:** Overview of samples used in this study.

ID	Gender	Age	Type of glioma	Grade	Type ofexperiment
S1	Male	40	astrocytoma	II	Chip
S2	Female	13	diffuse astrocytoma	II	Chip
S3	Male	37	diffuse astrocytoma	II	Chip
S4	Male	44	diffuse astrocytoma	II	Chip
S5	Female	33	diffuse astrocytoma	II	Chip
S6	Male	48	diffuse astrocytoma	II	Chip
S7	Female	32	medulloblastoma	II	Chip
S8	Female	63	oligodendroglioma	II	Chip
S9	Male	55	oligodendroglioma	II	Chip
S10	Male	37	anaplasia oligodendroglioma	III	Chip
S11	Female	55	anaplastic astrocytoma	III	Chip
S12	Male	57	anaplastic astrocytoma	III	Chip
S13	Female	43	anaplastic astrocytoma	III	Chip
S14	Male	39	anaplastic astrocytoma	III	Chip
S15	Female	55	astrocytoma	III	Chip
S16	Female	41	glioblastoma	IV	Chip
S17	Female	55	glioblastoma	IV	Chip
S18	Male	44	mixed oligodendroglioma	IV	Chip
S19	Male	39	anaplasia oligodendroglioma	II	RT-qPCR
S20	Male	7	medulloblastoma	III	RT-qPCR
S21	Male	11	glioblastoma	IV	RT-qPCR
S22	Female	59	glioblastoma	IV	RT-qPCR

Note: RT-qPCR: real-time quantitative PCR.

### Data Analysis and Functional Annotation

Illumina’s KaryoStudio (V1.0.3), together with the cnvPartition algorithm, was adopted to identify CNV regions. Illumina’s GenomeStudio software (V2009.1) with LOHscore plug-in was used to discover copy neutral loss of heterozygosity (cnLOH) regions. The samples were divided into LGG and HGG, and the analysis was limited to autosomal regions only due to the experiment design. The data used in this study were submitted to GEO with an accession of GSE34888.

GeneCoDis3 was used to relate genes to the corresponding Gene Ontology (GO) terms with cut-off = 0.05 [46], and WebGestalt V2 was used to map genes onto significant KEGG pathways with cut-off = 0.1 [47]. OMIM (Online Mendelian Inheritance in Man) was used to find relationship between genes and diseases [48].

### Real-time qPCR-based Validation Experiment

Four RNA samples, reverse-transcribed (Table 1), were subjected to real-time qPCR in a final reaction volume of 10 µl, which contains: 1 µl of 10×buffer, 0.25 µl of dNTP, 2 µl of gene-specific forward and reverse primer mix (Table 2), 1 µl cDNA (100 ng/µl), 0.1 µl of rTaq, 0.5 µl of fluorochrome (TIANGEN BIOTECH) and 5.15 µl of ddH2O.

**Table 2:** Primers and results of real-time qPCRs.

Gene	Primer	Product size (bp)	Fold change
AASS	F: AGCGCTACATAAGTCGTT	197	1.86
	R: GTCACATATTGCCACGAGTTT		
CYP2J2	F: AAAGAAGCCCTTATCCAC	187	30.53
	R: GAATGCGTTCCTCTAAGCTCT		
CYP4A11	F: CCTGGACCAGATGCCCTACAC	107	111.92
	R: CAGGGAAGGTGACGGGAGT		
PLA2G2A	F: CATGATCTTTGGCCTACTGC	108	6.57
	R: CAGCCGTAGAAGCCATAACTG		
PLA2G5	F: ACATTCGCACACAGTCCTAC	170	24.23
	R: GAGGATGTTGGGAAAGTATTG		
PTEN	F: CAGAAAGACTTGAAGGCGTAT	124	52.53
	R: TTGGCGGTGTCATAATGT		
RB1	F: TCCTCGAAGCCCTTACAAGTT	176	6.38
	R: TCTCAGAAGTCCCGAATGAT		

Note: These primers were designed by using Oligo6. Fold changes were computed by comparing between HGG and LGG.

## Results

### Case and Control

Using 18 solid tumors from patients (9 as LGG and 9 as HGG) as the case sample and the patients’ DNA isolated from their peripheral blood as the control, we compared chromosome aberrations between the case and control based on the Illumina’s BeadChip platform that defines both copy number variation (CNVs) and copy neutral loss of heterozygosity (cnLOH). This technology allows us to find sequence differences between tumor and blood samples at cytogenetic and molecular levels, which include both gains and losses. We only discovered sequence amplifications in the tumor not in the corresponding periphery blood. In other three aberration categories of CNVs, there are always more variations in the tumor than in the control, and the significance of variations between the paired samples can be evaluated statistically (P-value<0.05, paired t-test) when the total length influenced by CNVs in each sample was considered.

### CNVs among Autosomal Arms

After the survey of different CNVs in 44 autosomal arms in LGG and HGG, we removed those shared by tumors and their controls to identify tumor-specific variations. We made a few interesting observations. First, 13q harbors a major high-frequency genomic aberration class. For instance, there are 3, 3, 3, 2, and 2 samples found to have homozygous deletion, hemizygous deletion, cnLOHs, duplication and amplification, respectively. Second, we found that 22, 29, 38, 38, and 6 chromosomal arms have variations in the five categories, respectively. Obviously, cnLOH is one of the most frequent categories, and our results agreed with a previous report [43]. In addition, the skewed distribution of sequence amplification showed that there were more copy number gains found on only a few chromosomal arms. Third, we paid more attention to CNVs between LGG and HGG and found several homozygous deletions of 13q in HGG but not in LGG. There were also cnLOHs in three HGG, such as those on 15q and 17q, but none was found in LGG. In addition, four HGG had duplications on 3q but none was found in LGG.

### Comparison of LGG and HGG at the Cytoband Level

To pinpoint the position of variations on chromosomes, we investigated CNV and cnLOH in LGG and HGG. To reduce false positives, we only referred to those that occurred in at least two tumor samples (four samples for “duplications” category) after removing what appeared in the controls, i.e., corresponding blood samples (Table 3). Taking homozygous deletion as an example, we found several events on 8p11.23 in LGG, and 13q12.11 in HGG. In both hemizygous deletion and cnLOH, we found more cytobands in HGG than in LGG. Surprisingly, we found more duplication events in LGG than in HGG. Taken together, we conjectured that losses and gains occur more often in HGG and LGG, respectively. Furthermore, we found that gains usually occurred on 7q and 1p in HGG and LGG, respectively. Decreasing copy number is usually discovered on 6q, 13q and 19q in HGG. We also noticed that there were overlaps between the two tumor grades in the cnLOH category, especially the fact that cnLOHs were spread out more broadly in cross-chromosome cytobanding than the rest of copy number variation categories.

**Table 3:** Comparison of sample numbers of genomic aberration in gliomas at the cytoband level.

Homozygousdeletions	L	8p11.23	2				
	H	13q12.11	2				
Hemizygous deletions	L	5q14.3	2	11q11	2		
	H	6q12	2	6q13	2	6q14.1	2
	H	6q14.2	2	6q14.3	2	6q16.3	2
	H	6q21	2	6q22.1	2	6q22.31	2
	H	6q22.32	2	6q22.33	2	6q23.1	2
	H	6q23.2	2	6q23.3	2	6q24.1	2
	H	6q24.2	2	11p15.4	2	13q12.2	2
	H	13q12.3	2	13q13.1	2	13q13.2	2
	H	13q14.12	2	13q14.13	2	13q14.2	2
	H	13q14.3	2	13q21.1	2	13q21.2	2
	H	13q21.31	2	19q12	2	19q13.11	2
	H	19q13.12	2				
cnLOHs	L	2p12	2	6p21.32	2	6p21.33	2
	L	6p22.1	2	6q15	2	9p24.1	2
	L	22q12.3	2				
	H	2p23.2	2	2p25.1	3	2p25.3	2
	H	3q26.1	2	6p21.32	3	6p21.33	3
	H	6q25.1	2	8q22.3	2	8q24.3	3
	H	9p24.1	2	9p24.2	2	10p13	2
	H	10p15.1	2	14q32.13	2	15q26.1	2
	H	17p13.3	2	17q22	2	18q22.1	2
	H	18q22.3	2	18q23	2	21q22.3	2
	H	22q13.31	3				
Duplications	L	1p13.3	4	1p21.1	4	1p21.3	4
	L	1p22.2	4	1p22.3	4	1p31.1	4
	L	1p31.3	4	1p32.1	4	1p32.2	4
	L	1p32.3	4	1p33	4	1p34.3	4
	L	1p35.1	4	1p35.2	4	1p35.3	4
	L	1p36.11	4	1p36.12	4	1p36.13	4
	L	1p36.31	4				
	H	5p15.33	4	7q31.31	4	7q31.32	4
	H	7q31.33	4	7q33	4	7q34	4
	H	7q35	4	7q36.1	4	20q11.1	4
Amplifications	H	7p11.2	2				

Note: L and H stand for LGG and HGG, respectively.

### Genes Found to Associate with Gliomas

To identify specific genes associated with gliomas, we pooled all genomic aberrations occurred in at least six tumor samples. After filtered our data based on known variations found in the controls and Database of Genomic Variants (hg18.v8) (http://projects.tcag.ca/variation/), we had 24 genes and the related information was summarized (chromosome location, aberration category, tumor grading) (Table 4). These genes are all clustered on 1p, 7q, and 19q. Among them, 17 genes are only gains, and three of other genes, *VN1R2*, *VN1R4* and *ZNF677*, have all three types of genomic aberrations–gain, loss and cnLOH. Referencing to the annotation of the OMIM Morbid Map (http://www.ncbi.nlm.nih.gov/omim), we found that *AASS*, *TAS2R16* and *TSPAN12* are previously identified to be disease-related and associated with “*hyperlysinemia*”, “*alcohol dependence*” and “*exudative vitreoretinopathy*”, respectively.

**Table 4:** Selected genes involved in genomic aberration of gliomas.

Gene	Description	Location	Total	Gain	Loss	cnLOH	LGG	HGG
NKAIN1	Na+/K+ transporting ATPase interacting 1	1p	6	5	1		4	2
PTPRU	Protein tyrosine phosphatase, receptor type, U	1p	6	5	1		4	2
SLC44A3	Solute carrier family 44, member 3	1p	6	5	1		4	2
AASS	Aminoadipate-semialdehyde synthase	7q	6	6			2	4
ASB15	Ankyrin repeat and SOCS box-containing 15	7q	6	6			2	4
C7orf58	chromosome 7 open reading frame 58	7q	6	6			2	4
FEZF1	FEZ family zinc finger 1	7q	6	6			2	4
GPR37	G protein-coupled receptor 37 (endothelin receptor type B-like)	7q	6	6			2	4
HYAL4	Hyaluronoglucosaminidase 4	7q	6	6			2	4
ING3	Inhibitor of growth family, member 3	7q	6	6			2	4
IQUB	IQ motif and ubiquitin domain containing	7q	6	6			2	4
LMOD2	Leiomodin 2 (cardiac)	7q	6	6			2	4
NDUFA5	NADH dehydrogenase (ubiquinone) 1 alpha subcomplex, 5, 13 kDa	7q	6	6			2	4
POT1	POT1 protection of telomeres 1 homolog (S. pombe)	7q	6	6			2	4
RNF133	Ring finger protein 133	7q	6	6			2	4
RNF148	Ring finger protein 148	7q	6	6			2	4
SPAM1	Sperm adhesion molecule 1	7q	6	6			2	4
TAS2R16	Taste receptor, type 2, member 16	7q	6	6			2	4
TSPAN12	Tetraspanin 12	7q	6	6			2	4
WASL	Wiskott-Aldrich syndrome-like	7q	6	6			2	4
VN1R2	Vomeronasal 1 receptor 2	19q	6	4	1	1	5	1
VN1R4	Vomeronasal 1 receptor 4	19q	6	4	1	1	5	1
ZNF507	Zinc finger protein 507	19q	6	3	3		4	2
ZNF677	Zinc finger protein 677	19q	6	4	1	1	5	1

### Pathway Enrichment and Functional Annotation of the Associated Genes

To decipher functional relevance of genes and gene classification related to gliomas, we established datasets based on the following two criteria: (i) elimination of genes shared by both the case and the control and (ii) removal of genes shared by less than four samples. Altogether, we collected 442 LGG and 111 HGG associated genes. After mapping the two groups of genes onto the KEGG pathways, we obtained 12 pathways for LGG, which belong to “lipid metabolism” (in the number of pathways: 6), “neurodegenerative diseases” (1), “endocrine system” (1), “nervous system” (1), “circulatory system” (1), “signal transduction” (1), and “immune system” (1) (Table 5). These genes were categorized in 10 pathways correlated to HGG, among which two were classified as “lipid metabolism” and others were distributed in diverse classifications such as “signaling molecules and interaction”, “signal transduction”, “endocrine system”, “carbohydrate metabolism”, “amino acid metabolism”, “glycan biosynthesis and metabolism”, and “infectious diseases”. The most four significant pathways identified in LGG are “*arachidonic acid metabolism*”, “*linoleic acid metabolism*”, “*alpha-Linolenic acid metabolism*”, and “*ether lipid metabolism*”. In HGG, the most four enriched terms are “*metabolic pathways*”, “*neuroactive ligand-receptor interaction*”, “*calcium signaling pathway*”, and “*melanogenesis*”. We found 12 and 4 enriched GO terms associated with LGG and HGG, respectively (Table 6). In LGG, the top two GO terms were “*DNA binding*”, and “*regulation of transcription, DNA-dependent*”. In HGG, the top two GO terms were “*neuronal cell body*”, and “*defense response to bacterium*”. To our current knowledge, tumors often alter cellular processes, such as proliferation, growth, programmed death, differentiation, division, mutation-induced DNA damage, and repair [1]. All these newly discovered distinct function categories may introduce features of primary (low-grade) or malignant (high-grade) tumors.

**Table 5:** Pathway analysis of genes involved in genomic aberration in LGG (A) and HGG (B).

(A) LGG				
Pathway name	Observed number	Expected number	R	FDR
Arachidonic acid metabolism	9	1.35	6.69	1.00E−04
Linoleic acid metabolism	7	0.67	10.41	1.00E−04
alpha-Linolenic acid metabolism	6	0.43	13.87	1.00E−04
Ether lipid metabolism	7	0.82	8.57	2.00E−04
Glycerophospholipid metabolism	8	1.61	4.97	2.10E−03
Prion diseases	6	0.84	7.14	2.10E−03
GnRH signaling pathway	9	2.38	3.78	5.50E−03
Long-term depression	7	1.63	4.28	9.60E−03
Vascular smooth muscle contraction	9	2.71	3.32	1.14E−02
VEGF signaling pathway	7	1.78	3.94	1.28E−02
Fc epsilon RI signaling pathway	7	1.85	3.78	1.45E−02
Fatty acid metabolism	4	1.01	3.96	9.55E−02
**(B) HGG**				
**Pathway name**	**Observed number**	**Expected number**	**R**	**FDR**
Metabolic pathways	14	6.21	2.25	6.00E−02
Neuroactive ligand-receptor interaction	5	1.48	3.37	6.00E−02
Calcium signaling pathway	4	1.02	3.92	6.00E−02
Melanogenesis	3	0.58	5.14	6.00E−02
Fructose and mannose metabolism	2	0.2	10.08	6.00E−02
Lysine degradation	2	0.25	7.97	6.00E−02
Androgen and estrogen metabolism	2	0.26	7.62	6.00E−02
Glycerolipid metabolism	2	0.25	7.97	6.00E−02
Glycosaminoglycan degradation	2	0.12	16.32	6.00E−02
Vibrio cholerae infection	2	0.31	6.47	7.28E−02

Note: R indicates the ratio of enrichment.

**Table 6:** GO enrichment analysis of genes involved in genomic aberration in LGG (A) and HGG (B).

(A) LGG				
GO term	Description	NG	NGR	Hyp*
GO:0003677	DNA binding (MF)	104	1651	5.89E−18
GO:0006355	regulation of transcription, DNA-dependent (BP)	95	1473	2.27E−16
GO:0008270	zinc ion binding (MF)	103	1780	2.16E−15
GO:0005622	intracellular (CC)	100	1774	4.42E−14
GO:0046872	metal ion binding (MF)	120	2649	1.39E−10
GO:0003676	nucleic acid binding (MF)	42	721	1.11E−05
GO:0006644	phospholipid metabolic process (BP)	8	31	2.35E−04
GO:0016503	pheromone receptor activity (MF)	3	3	9.58E−04
GO:0019236	response to pheromone (BP)	3	3	4.16E−03
GO:0004623	phospholipase A2 activity (MF)	5	20	5.27E−03
GO:0005634	nucleus (CC)	156	4968	6.35E−03
GO:0019205	nucleobase-containing compound kinase activity (MF)	3	7	2.23E−02
**(B) HGG**				
**GO term**	**Description**	**NG**	**NGR**	Hyp*
GO:0043025	neuronal cell body (CC)	9	205	4.00E−04
GO:0042742	defense response to bacterium (BP)	6	81	3.41E−03
GO:0015382	sodium:sulfate symporter activity (MF)	2	2	5.95E−03
GO:0004415	hyalurononglucosaminidase activity (MF)	2	6	4.39E−02

Note:

NG = Number of annotated genes in the inquired list.

NGR = Number of annotated genes in the reference list.

Hyp* = Corrected hypergeometric P-value.

### Correlating Copy Number Variation with Gene Expression

We used real-time qPCR to validate the expression of seven identified genes: *AASS*, *CYP2J2*, *CYP4A11*, *PLA2G2A*, *PLA2G5*, *PTEN,* and *RB1*. First, all genes showed increased expression when compared in the two tumor grades (HGG vs. LGG) (Table 2). *CYP2J2*, *CYP4A11*, *PLA2G5* and *PTEN* exhibited even over 10 fold changes. We did not compare the gene expressions between the tumors and the controls, their corresponding blood cells, due to the tissue-specific nature of the gene expression. Of the seven genes, we noticed *RB1* gains in HGG and losses in both grades. Other different aberrations include (1) *CYP2J2*, *CYP4A11*, *PLA2G2A*, and *PLA2G5* gains in LGG, (2) *AASS* gain in both, and (3) *PTEN* gains in HGG only. The correlation between expression and copy number is complex, and has been demonstrated to be positive under most conditions with several exceptions of negative correlations [12]. There are a few interpretations for the complicated relations between gene (or segment) copy number and gene expression. First, the higher gene dosage leads to an increased transcript production and a positive correlation. Second, negative correlation has been proposed that some possible compensation or time-series feedback effects may act on the progression or cell response of tumors [12]. Last, a handful of CNVs can destroy regulatory regions to some extents and inhibit the expression of relevant genes. In any case, we have found significant differential gene expressions in selected genomic structures and genes between LGG and HGG.

## Discussion

In accordance with Knudson’s two-hit hypothesis on tumor formation [49], tumorigenesis occurs concurrently with activation of oncogenes and inactivation of tumor suppressor genes (TSGs). In particular, tumor formation is a process where the potential for malignancy increases with mutation accumulation [50]. Our results support the concept that genomes in HGG tend to be deleted more intensively than those in LGG at both cytoband and molecular levels. In other words, there are gains of genes or partial sequences in the primary stage of the tumors and subsequently losses of tumor suppressor genes or TSGs appear to escape from normal cellular controls. For instance, cnLOHs in HGG happened in twelve chromosomes (2, 3, 6, 8, 9, 10, 14, 15, 17, 18, 21, and 22). In contrast, cnLOHs in LGG occurred only in four chromosomes (2, 6, 9, and 22) (Table 3). This particular clear observation in cnLOHs has not been found in other variation types, and the result indicates that disappearance of heterozygosity and allelic losses together with gene functions are major contributors to HGG.

We have effectively validated many locations, genes, pathways, and function categories in keeping with the previous studies at different levels. At the chromosome level, among the five variation classes, cnLOH is the major type, which is distributed over all chromosomes. Furthermore, we have validated many variations at the cytoband level (Table 3). For instance, we found duplications on 7q31.31, 7q31.32, 7q31.33, 7q33, 7q34, 7q35 and 7q36.1 in HGG, which is in agreement with gains of 7q reported in both grades [37]. We discovered hemizygous deletions on numerous cytobands (e.g., 6q12, 6q13, 6q14.1, 6q16.3, 6q21, 6q22.1, 6q23.1, 6q24.1, 13q12.2, 13q13.1, 13q14.12, 13q21.1, 19q12, and 19q13.11) in line with losses of 6q, 13q, and 19q in HGG [37], [44]. There have been some contradicting results in this study; for example, we only identified duplications on 1p in LGG and hemizygous deletions on 19q in HGG but LOHs on 1p-19q were reported in both grades by another group [41]. We also have some novel findings specific to Chinese populations and observed the complicated and altered roles of traditional tumor-related genes in our study. For example, *KIT* gains in 2 LGG and 2 HGG, *MGMT* gains in 1 LGG and 3 HGG, *MXI1* gains in 1 LGG and 3 HGG, *DMBT1* gains in 1 LGG and 3 HGG, *IDH1* only gains in one LGG. Thus, TSGs and oncogenes are relative and condition-sensitive concept, i.e., TSGs in one sample may be oncogenes in another. We finally inferred that TSGs not only can be lost but also can be gained, whereas oncogenes appear generally being gained in these tumors.

We did not find any obvious major pathways shared by LGG and HGG but identified 6 and 2 subclasses from the major class “*lipid metabolism*” shared by the two grades, respectively. In HGG, we noticed some interesting variations, including *AASS* (4 gains) in “*lysine degradation*” and *CHRM2* (4 gains), *CYSLTR2* (2 gains and 2 losses), *GRM8* (4 gains), *HTR2A* (2 gains and 2 losses), and *MLNR* (2 gains and 2 losses) in “*neuroactive ligand-receptor interaction*”. We further found variations of *GRM8* (2 gains) and *CYSLTR2* (1 loss), *HTR2A* (1 loss), *MLNR* (1 loss) in LGG. Gains in the four LGG are *CYP2J2*, *CYP4A11*, *CYP4A22*, *PLA2G2A*, *PLA2G2C*, *PLA2G2D*, *PLA2G2E*, *PLA2G2F*, and *PLA2G5,* and they are involved in “*arachidonic acid metabolism*”. Looking into well-known oncogenes and TSGs, we identified gains of *EGFR* in “*de novo glioma pathway*” (2 in LGG and 3 in HGG), as well as *MDM2* (1 in LGG and 3 in HGG) and *PTEN* (1 in HGG), and losses of *CDKN2A* (2 in HGG). In “*secondary glioma pathway*”, gains of *PDGFA* (1 in LGG and 2 in HGG), *PDGFRA* (2 in LGG and 2 in HGG) and *CDK4* (1 in LGG and 2 in HGG) and *RB1* (2 in HGG), and losses of *RB1* (1 in LGG and 2 in HGG) are also identified.

Our study was designed to uncover molecular differences between LGG and HGG, which are largely based on pathology, morphology, and degree of malignancy, and for which we know that altered genomic regions of HGG are more severe than those of LGG and that patient survival time is shorter in HGG (data not shown). Based on our study, we hope to associate genetics to clinical outcomes in three crucial aspects of fighting the disease, including diagnosis, treatment, and prognosis. First, specific and dominant genomic variations in cytobands or genes may be used to classify the two tumor grades at molecular level. Second, different genes with DNA aberrations may be further pursued as potential drug targets, especially in the personalized or population-specific cancer treatment for Chinese populations. Third, the disease-associated genes from LGG and HGG may be used for inferring survival time and guidance for disease treat [51], [52], [53]. In addition, we proposed a theory that losses of genes or alleles may play greater role in the formation and progression of HGG although both gains and losses contribute. This phenomenon is only observed at the cytoband level and relevant to concentrated alternations of homologous or heterogeneous genome composition [54]. Therefore, this is not a decisive but a substantial measure for glioma morbidity. Further large-scale investigations are needed for further validations.

**Figure 1:**
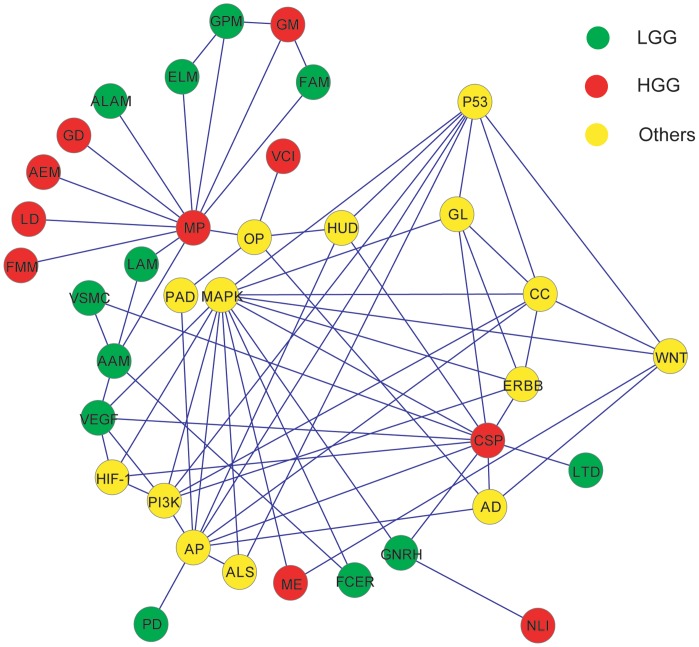
A network of pathways related to LGG, HGG, and others. The abbreviations for KEGG pathways used in this analysis are listed as follows. AAM: Arachidonic acid metabolism; LAM: Linoleic acid metabolism; ALAM: alpha-Linolenic acid metabolism; ELM: Ether lipid metabolism; GPM: Glycerophospholipid metabolism; PD: Prion diseases; GNRH: GnRH signaling pathway; LTD: Long-term depression; VSMC: Vascular smooth muscle contraction; VEGF: VEGF signaling pathway; FCER: Fc epsilon RI signaling pathway; FAM: Fatty acid metabolism; MP: Metabolic pathways; NLI: Neuroactive ligand-receptor interaction; CSP: Calcium signaling pathway; ME: Melanogenesis; FMM: Fructose and mannose metabolism; LD: Lysine degradation; AEM: Androgen and estrogen metabolism; GM: Glycerolipid metabolism; GD: Glycosaminoglycan degradation; VCI: Vibrio cholerae infection; AD: Alzheimer's disease; PAD: Parkinson's disease; ALS: Amyotrophic lateral sclerosis; HUD: Huntington's disease; PI3K: PI3K-Akt signaling pathway; HIF-1: HIF-1 signaling pathway; MAPK: MAPK signaling pathway; CC: Cell cycle; P53: p53 signaling pathway; AP: Apoptosis; GL: Glioma; ERBB: ErbB signaling pathway; WNT: Wnt signaling pathway; and OP: Oxidative phosphorylation.

Ultimately, we should be able to build an intricate network that is composed of pathways involved in gliomas both identified in this study and other known in the previous literature as we exemplified in Figure 1. The first characteristic of the network is the difficulty to fully discriminate all three complex modules of the pathways, such as LGG, HGG and others. However, we are able to identify major hubs among the modules, such as *MP* and *MAPK*, in the categories of HGG and others, respectively; some may even be obviously LGG associated, such as *AAM*. In addition, the tissue-specificity of gliomas is blurred by *AP* and other five vertices including *PD*, *ALS*, *AD*, *HUD* and *PAD*, which are common for neurodegenerative diseases and other neuro-pathological conditions. As a final note, the patterns of the three categories may suggest that there are differences in disease-causing mechanisms between Chinese and other populations. It is undoubted that studies on larger samples, coupled with in-depth analyses and validations, are what we should do in the near future.
